# Catalyst-Free Synthesis of Highly Biologically Active 5-Arylidene Rhodanine and 2,4-Thiazolidinedione Derivatives Using Aldonitrones in Polyethylene Glycol

**DOI:** 10.1155/2013/273534

**Published:** 2013-02-14

**Authors:** Dhruva Kumar, Suresh Narwal, Jagir S. Sandhu

**Affiliations:** Department of Chemistry, Punjabi University, Patiala, Punjab 147002, India

## Abstract

A green, efficient synthesis of 5-arylidene rhodanine and 2,4-thiazolidinedione derivatives without using any external catalyst in polyethylene glycol (PEG) at 80°C has been described. Reaction procedure is very simple, short, and obtained yields are very high.

## 1. Introduction

**Figure 1:**
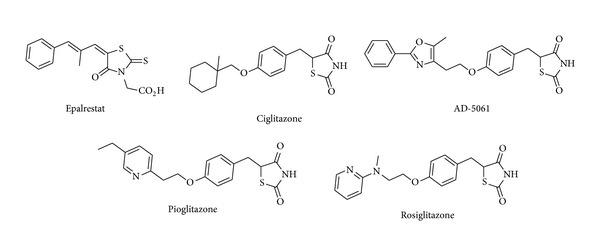
Clinically used molecules having 5-arylidene rhodanines and 2,4-thiazolidenediones.

Rhodanines and thiazolidinediones both are aprivileged class of molecule, and they show large number of biological activities. The most significant position of these molecules seems to be as they are asubset of commercially employed noninsulin-dependent diabetes mellitus (NIDDM), insulin sensitizing agents (Figure 1) such as epalrestat, ciglitazone, AD-5061, pioglitazone, rosiglitazone, and so forth.

Furthermore, rhodanine derivatives possess anticonvulsant, antibacterial, antiviral, and antidiabetic activities [1–3] Some of rhodanine-based derivatives act as hepatitis C virus (HCV) protease inhibitor [4], uridine diphospho-*N*-acetylmuramate/L-alanine ligase inhibitor [5], aldose reductase [6], *β*-lactamase [7], and JNK-stimulating phosphatase-1 (JSP-1) [8], while some of its derivatives are used for the analysis of certain noble metal ions [9]. Therefore, the synthesis of rhodanine derivatives currently is of much importance and a variety of methods and catalysts have been used [10–12].

Unlike rhodanine, 2,4-thiazolidinedione derivatives also have remarkable biological activities like antidiabetic [13], antibacterial [14], antifungal [15], antiproliferative effect on vascular smooth muscle [16], aldose reductase inhibitors [17], 15-hydroxyprostaglandin dehydrogenase inhibitors [18] instead of these biological activities 5-benzylidene-thiazolidine-2,4-dione derivatives act as inhibitors of MurD ligase [19]. In the literature several methods have been reported to synthesize these privileged molecules [20–24]. Therefore, significant biological activities prompt us to synthesize thiozolidine derivatives. 

**Scheme 1:**
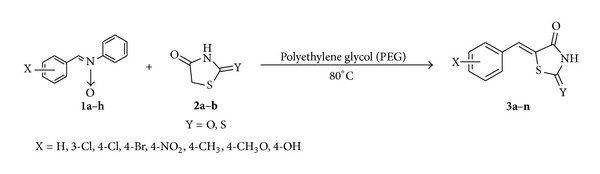
Synthesis of 5-arylidene thiazolidinediones.

Nitrones (imine oxides) are reputed as 1,3-dipoles and are extensively explored for the synthesis of five membered heterocycles by combining them with several types of multiple bonds [25–27]. Apart from this major utility their general chemistry is little studied.[27] There are few reports of successful 1,3-additions of nitrones [28, 29]. Yousif et al. reported reactions of heterocyclic N-oxides under acidic conditions and obtained only condensed products [30]. In contrast, their counterpart imines are extensively explored to expose their utility as aldehyde equivalent [31–33]. Present protocol is the environment benign synthesis of 5-arylidene rhodanine and 2,4-thiazolidinedione derivatives using aldonitrones in polyethylene glycol (PEG). The reaction proceeds via addition-elimination way and afforded the desire products in very good to excellent yield (Scheme 1).

## 2. Results and Discussion

First of all, a series of nitrones was prepared using a variety of aldehyde and hydroxyl amine as per already reported method [34]. A mixture of freshly prepared N-phenyl-N-phenylmethylidenamine oxide (10 mmol) **1a** and rhodanine (10 mmol) **2a** was stirred at 80°C in polyethylene glycol (PEG) for 20 min to afford corresponding arylidene rhodanine **3a** *via* addition-elimination process.

To check the effect of the solvent, a set of reactions was performed using different solvents such as MeOH, EtOH, H_2_O, THF, PEG, DMF, and so forth and in absence of solvent. Conclusively, in case of PEG best results were obtained with high yields in minimum reduced time. Keeping optimized reaction conditions, a variety of aldehydes with rhodanine/2,4-thiazolidinedione were reacted to afford 5-arylidenerhodanines **3a–g** and 5-arylidene-2,4-thiazolidinediones **3h–n** with excellent yields (Table 1).

**Scheme 2:**
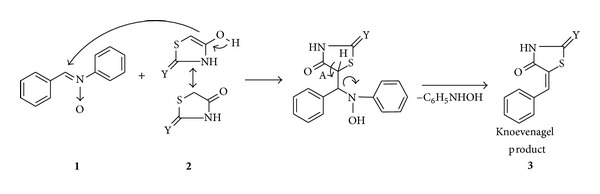
Plausible reaction mechanism.

Active methylene compounds **2a–b** afforded the Knoevenagel products **3a–n** selectively with exo-double bond without the formation of other side-products/bis-products as shown in Scheme 2 via addition-elimination process. Electron withdrawing and donating groups on aromatic *N*-oxides showd slightly diversion in rate of reaction and yields; that is, electron withdrawing group containing aromatic *N*-oxides afforded arylidene compounds with better yields in shorter reaction time (Table 1). 

Next, the recyclability of the solvent was studied by using **1a** and **2a** as the model substrates. We observed that PEG could be recovered by under vacuum filtration of products obtained on cooling. PEG recovered as filtrate and was successfully recycled and reused for five runs.

As far as mechanism is concerned, reaction proceeds via nucleophilic addition of **2** on **1** with subsequent elimination of amine part of aromatic N-oxides to afford arylidene products **3** (Scheme 2). 

## 3. Conclusion

In summary, the present protocol is an efficient and environmentally benign procedure for the synthesis of drug intermediate 5-arylidine rhodanine and 2,4-thiazolidinedione derivatives using aldonitrones in polyethylene glycol (PEG) *via* simple addition-elimination process. Present protocol does not need any external catalyst, and it is applicable on a variety of nitrones. This method produces good to excellent yields in shorter reaction time, and it seems that reaction is autocatalyzed because eliminatingpart of nitrone acts as catalyst.

## 4. Experimental Section

### 4.1. General

Reagent-grade chemicals were purchased from a commercial source and used without further purification. Yields refer to the yield of the isolated products. Melting points were determined in open capillaries in paraffin bath and are uncorrected. Infrared (IR) spectra were recorded in KBr discs on a Perkin-Elmer 240C analyzer. ^1^H NMR spectra were recorded on a BRUKER AVANCE II 400 NMR Spectrometer using tetramethylsilane (TMS) as internal standard. The progress of the reaction was monitored by thin layer chromatography (TLC) using silica gel G (Merck). 

### 4.2. General Method for the Synthesis of 5-Arylidene Rhodanine and 2,4-Thiazolidinedione (**3a–n**)

A mixture-nitrone (10 mmol) 1, rhodanine or 2,4-thiazolidinedione (10 mmol) 2, and polyethylene glycol (5 mL) was stirred at 80°C temperature for appropriate time (see Table 1). Reaction progress was monitored via TLC. After reaction completion, crude product was precipitated out on cooling. Obtained product was filtered, dried, and for further purification recrystallized from ethanol-DMF.

### 4.3. Spectral Data of Reprehensive Compounds

(5Z)-5-Benzylidene-2-thioxo-1,3-thiazolidine-4-one (**3a**): IR (KBr): 3390, 1709, 1669, 1600, 1429, 1200 cm^−1^. ^1^H NMR (300 MHz, DMSO-d_6_) *δ*_H_: 13.57 (s, 1H, NH), 7.68 (s, 1H, =CH), 7.48–7.79 (m, 5H, Ar–H). 

(5Z)-5-(4-Methoxybenzylidene)-2-thioxo-1,3-thiazolidine-4-one (**3f**): ^1^H NMR (300 MHz, DMSO-d_6_) *δ*_H_: 13.71 (s, 1H, NH), 7.61 (s, 1H, =CH), 7.52 (d, 2H, *j *= 8.2 Hz, Ar–H), 7.08 (d, 2H, *j *= 8.2 Hz, Ar–H), 3.09 (s, 3H, OCH_3_).

(5Z)-5-Benzylidene-1,3-thiazolidine-2,4-dione **(3h):** IR (KBr) cm^−1^:  3155 (NH), 3049, 879 (CH; aromatic), 2868 (CH; aliphatic), 1739, 1691 (C=O). ^1^H NMR (300 MHz, DMSO-d_6_) *δ*_H_: 8.27 (1H, s, NH), 7.86 (1H, s, CH), 7.26 (5H, m, aromatic protons). MS *m*/*z* (%): 206 (*M* + 1).

 (5Z)-5-(4-Methylbenzylidene)-1,3-thiazolidine-2,4-dione (**3m**): IR (KBr) 3211, 1725, 1689 cm^−1^. ^1^H NMR (300 MHz, DMSO-d_6_) *δ*_H_: 12.50 (s, 1H, br, NH), 7.69 (s, 1H, =CH), 7.50 (d, 2H, *j *= 8.3 Hz, Ar–H), 7.11 (d, 2H, *j *= 8.3 Hz, Ar–H), 3.49 (s, 3H, OCH_3_).

## Figures and Tables

**Table 1:** Synthesis of 5-arylidine rhodanine and 2,4-thiazolidinedione derivatives using aldonitrones in polyethylene glycol (PEG).

Entry	X	Y	Product^a^	Time (min)	Yield (%)^b^	Melting point (°C) reference
1	H	S	**3a**	20	85	202-203 [11]
2	4-Cl	S	**3b**	20	93	230–232 [11]
3	4-Br	S	**3c**	20	89	229-230 [3]
4	4-NO_2_	S	**3d**	25	94	254-255 [11]
5	4-CH_3_	S	**3e**	30	78	224-225 [11]
6	4-CH_3_O	S	**3f**	30	83	250-251 [11]
7	4-OH	S	**3g**	30	79	184-185 [35]
8	H	O	**3h**	25	92	240-241 [36]
9	3-Cl	O	**3i**	25	88	270-271 [36]
10	4-Cl	O	**3j**	25	85	224-225 [37]
11	4-NO_2_	O	**3k**	25	75	260–262 [37]
12	4-CH_3_	O	**3l**	25	86	224-225 [37]
13	4-CH_3_O	O	**3m**	35	88	234-235 [36]
14	4-OH	O	**3n**	35	90	280-281 [36]

^a^Reaction conditions: **1a**–**h** (10 mmol), **2a**-**b** (10 mmol), and polyethylene glycol (PEG) 5 mL were heated at 80°C on magnetic stirrer. The products were characterized by spectral techniques like IR, ^1^H NMR. ^b^Isolated yields after recrystallization.

## References

[1] Yu M, Kanji M, Hitoshi I, Chitoshi H, Satoru O, Takashi S (1991). Studies on antidiabetic agents—10. Synthesis and biological activities of pioglitazone and related compounds. *Chemical & Pharmaceutical Bulletin*.

[2] Ahn JH, Kim SJ, Park WS (2006). Synthesis and biological evaluation of rhodanine derivatives as PRL-3 inhibitors. *Bioorganic and Medicinal Chemistry Letters*.

[3] Sortino M, Delgado P, Juárez S (2007). Synthesis and antifungal activity of (Z)-5-arylidenerhodanines. *Bioorganic and Medicinal Chemistry*.

[4] Wan TS, Cheng LL, Su LY, Siew PL, Mui MS (2001). Arylalkylidene rhodanine with bulky and hydrophobic functional group as selective HCV NS3 protease inhibitor. *Bioorganic & Medicinal Chemistry Letters*.

[5] Sim MM, Ng SB, Buss AD, Crasta SC, Goh KL, Lee SK (2002). Benzylidene rhodanines as novel inhibitors of UDP-N-acetylmuramate/L-alanine ligase. *Bioorganic & Medicinal Chemistry Letters*.

[6] MacCari R, Corso AD, Giglio M, Moschini R, Mura U, Ottan R (2011). In vitro evaluation of 5-arylidene-2-thioxo-4-thiazolidinones active as aldose reductase inhibitors. *Bioorganic & Medicinal Chemistry Letters*.

[7] Grant EB, Guiadeen D, Baum EZ (2000). The synthesis and SAR of rhodanines as novel class C *β*-lactamase inhibitors. *Bioorganic & Medicinal Chemistry Letters*.

[8] Neil SC, Christine O, Marina P (2005). Rhodanine derivatives as inhibitors of JSP-1. *Bioorganic & Medicinal Chemistry Letters*.

[9] Tang E, Yang G, Yin J (2003). Studies on the synthesis of 5-(p-aminobenzylidene)-rhodanine and its properties. *Spectrochimica Acta Part A*.

[10] Heerding DA, Christmann LT, Clark TJ (2003). New benzylidenethiazolidinediones as antibacterial agents. *Bioorganic and Medicinal Chemistry Letters*.

[11] Jian-Feng Z, Feng-Xia Z, Yuan-Zhi S, Yu-Lan Z (2006). Synthesis of 5-arylalkylidenerhodanines catalyzed by tetrabutylammonium bromine in water under microwave irradiation. *Archive for Organic Chemistry*.

[12] Lee CL, Sim MM (2000). Solid-phase combinatorial synthesis of 5-arylalkylidene rhodanine. *Tetrahedron Letters*.

[13] Cantello BCC, Cawthorne MA, Cottam GP (1994). [[*ω*-(Heterocyclylamino)alkoxy]benzyl]-2,4-thiazolidinediones as potent antihyperglycemic agents. *Journal of Medicinal Chemistry*.

[14] Labouta IM, Salama HM, Eshba NH, Kader O, El-chrbini E (1987). Potential anti-microbial: syntheses and *in vitro* anti-microbial evaluation of some 5-arylazo-thiazolidones and related compounds. *European Journal of Medicinal Chemistry*.

[15] Ottanà R, MacCari R, Barreca ML (2005). 5-Arylidene-2-imino-4-thiazolidinones: design and synthesis of novel anti-inflammatory agents. *Bioorganic and Medicinal Chemistry*.

[16] Peuler JD, Phare SM, Iannucci AR, Hodorek MJ (1996). Differential inhibitory effects of antidiabetic drugs on arterial smooth muscle cell proliferation. *American Journal of Hypertension*.

[17] Bruno G, Costantino L, Curinga C (2002). Synthesis and aldose reductase inhibitory activity of 5-arylidene-2,4-thiazolidinediones. *Bioorganic and Medicinal Chemistry*.

[18] Wu Y, Karna S, Choi CH (2011). Synthesis and biological evaluation of novel thiazolidinedione analogues as 15-hydroxyprostaglandin dehydrogenase inhibitors. *Journal of Medicinal Chemistry*.

[19] Nace Z, Tihomir T, Roman S (2010). Discovery of novel 5-benzylidenerhodanine and 5-benzylidenethiazolidine-2, 4-dione inhibitors of MurD ligase. *Journal of Medicinal Chemistry*.

[20] Ibrahim MA, Abdel-Hamed MAM, El-Gohary NM (2011). A new approach for the synthesis of bioactive heteroaryl thiazolidine-2,4-diones. *Journal of the Brazilian Chemical Society*.

[21] Mahalle S, Ligampalle D, Mane R (2009). Microwave-assisted synthesis of some 2,4-thiazolidinedione derivatives. *Heteroatom Chemistry*.

[22] Yang DH, Yang BY, Chen ZC, Chen SY (2006). A convenient synthesis of 5-arylidenethiazolidine-2,4-diones on potassium fluoride-aluminium oxide. *Organic Preparations and Procedures International*.

[23] Yang BY, Yang DH (2011). Solvent-free synthesis of 5-benzylidene-2-thioxothiazolidin-4-ones and thiazolidine-2,4-diones catalysed by glycine under microwave irradiation. *Journal of Chemical Research*.

[24] Shelke KF, Sapkal SB, Madje BR, Shingate BB, Shingare MS (2009). Ionic liquid promoted an efficient synthesis of 5-arylidene-2,4-thiazolidinedione. *Bulletin of the Catalysis Society of India*.

[25] Padwa A (1984). *1,3-Dipolar Cycloaddition Chemistry*.

[26] Torssell KBG (1988). *Nitrile Oxides, Nitrones, and Nitronate in Organic Synthesis*.

[27] Banerji A, Sengupta P (2001). Recent studies on 1,3-dipolar cycloadditions of nitrones. *Journal of the Indian Institute of Science*.

[28] Okino T, Hoashi Y, Takemoto Y (2003). Thiourea-catalyzed nucleophilic addition of TMSCN and ketene silyl acetals to nitrones and aldehydes. *Tetrahedron Letters*.

[29] Murahashi SI, Imada Y, Kawakami T, Harada K, Yonemushi Y, Tomita N (2002). Enantioselective addition of ketene silyl acetals to nitrones catalyzed by chiral titanium complexes. Synthesis of optically active *β*-amino acids. *Journal of the American Chemical Society*.

[30] Yousif MM, Seitaro S, Masatomo H (1982). Studies on tertiary amine oxides—72. Reactions of aromatic N-oxides with Meldrum's acid in the presence of acetic anhydride. *Chemical & Pharmaceutical Bulletin*.

[31] Chiba R, Oriyama T (2008). A highly stereoselective knoevenagel reaction of N-tosylimines with active methylene compounds in DMSO. *Chemistry Letters*.

[32] Ramadan AM, Khadijah MA, Asma A, Kamal US (2011). Solar thermochemical reactions—4: unusual reaction of nitrones with acetonitrile derivatives induced by solar thermal energy. *Green and Sustainable Chemistry*.

[33] Patai S (1970). *The Chemistry of the Carbon-Nitrogen Double Bond*.

[34] Jan H, Anthony M (1964). Nitrones. *Chemical Reviews*.

[35] Gong K, He Z-W, Xu Y, Fang D, Liu Z-L (2008). Green synthesis of 5-benzylidene rhodanine derivatives catalyzed by 1-butyl-3-methyl imidazolium hydroxide in water. *Monatshefte für Chemie*.

[36] Shelke KF, Sapkal SB, Madje BR, Shingate BB, Shingare MS (2009). Ionic liquid promoted an efficient synthesis of 5-arylidene-2,4-thiazolidinedione. *Bulletin of the Catalysis Society of India*.

[37] Zhang Y, Zhou Z (2012). A solvent-free protocol for the green synthesis of 5-arylidene-2,4-thiazolidinediones using ethylenediamine diacetate as catalyst. *Organic Chemistry International*.

